# Color vision evolution in egg-laying mammals: insights from visual photoreceptors and daily activities of Australian echidnas

**DOI:** 10.1186/s40851-023-00224-7

**Published:** 2024-01-02

**Authors:** Shiina Sakamoto, Yuka Matsushita, Akihiro Itoigawa, Takumi Ezawa, Takeshi Fujitani, Kenichiro Takakura, Yang Zhou, Guojie Zhang, Frank Grutzner, Shoji Kawamura, Takashi Hayakawa

**Affiliations:** 1 Graduate School of Environmental Science, Hokkaido University, Sapporo, Hokkaido Japan; 2 Department of Integrated Biosciences, Graduate School of Frontier Sciences, The University of Tokyo, Kashiwa, Chiba Japan; 3 Department of Agricultural Chemistry, School of Agriculture, Meiji University, Kawasaki, Kanagawa Japan; 4 Japan Society for the Promotion of Science, Tokyo, Japan; 5 Nagoya Higashiyama Zoo, Nagoya, Aichi Japan; 6 BGI Research, Shenzhen, China; 7 BGI Research, Wuhan, China; 8 Center of Evolutionary & Organismal Biology, Zhejiang University School of Medicine, Hangzhou, China; 9 The Environment Institute, University of Adelaide, Adelaide, SA Australia; 10 Faculty of Environmental Earth Science, Hokkaido University, Sapporo, Hokkaido Japan

**Keywords:** Color vision, Opsin, LWS, SWS2, *λ*_max_, Monotreme, Echidna, Platypus, Cathemerality, Ethogram

## Abstract

Egg-laying mammals (monotremes) are considered “primitive” due to traits such as oviparity, cloaca, and incomplete homeothermy, all of which they share with reptiles. Two groups of monotremes, the terrestrial echidna (Tachyglossidae) and semiaquatic platypus (Ornithorhynchidae), have evolved highly divergent characters since their emergence in the Cenozoic era. These evolutionary differences, notably including distinct electrosensory and chemosensory systems, result from adaptations to species-specific habitat conditions. To date, very few studies have examined the visual adaptation of echidna and platypus. In the present study, we show that echidna and platypus have different light absorption spectra in their dichromatic visual sensory systems at the molecular level. We analyzed absorption spectra of monotreme color opsins, long-wavelength sensitive opsin (LWS) and short-wavelength sensitive opsin 2 (SWS2). The wavelength of maximum absorbance (*λ*_max_) in LWS was 570.2 in short-beaked echidna (*Tachyglossus aculeatus*) and 560.6 nm in platypus (*Ornithorhynchus anatinus*); in SWS2, *λ*_max_ was 451.7 and 442.6 nm, respectively. Thus, the spectral range in echidna color vision is ~ 10 nm longer overall than in platypus. Natural selection analysis showed that the molecular evolution of monotreme color opsins is generally functionally conserved, suggesting that these taxa rely on species-specific color vision. In order to understand the usage of color vision in monotremes, we made 24-h behavioral observations of captive echidnas at warm temperatures and analyzed the resultant ethograms. Echidnas showed cathemeral activity and various behavioral repertoires such as feeding, traveling, digging, and self-grooming without light/dark environment selectivity. Halting (careful) behavior is more frequent in dark conditions, which suggests that echidnas may be more dependent on vision during the day and olfaction at night. Color vision functions have contributed to dynamic adaptations and dramatic ecological changes during the ~ 60 million years of divergent monotreme evolution. The ethogram of various day and night behaviors in captive echidnas also contributes information relevant to habitat conservation and animal welfare in this iconic species, which is locally endangered.

**Supplementary Information:**

The online version contains supplementary material available at 10.1186/s40851-023-00224-7.

## Background

### Vertebrate visual pigments

Animals evolved senses to adapt traits conferring fitness advantages in various habitats specific to species and populations, such as diet selection, predator avoidance, mate choice, and intraspecific communication. The visual sensory system provides environmental light spectra recognition and discrimination. Particularly, color vision is important information in vision-oriented animals [1]. Basal vertebrates presumably had a tetrachromatic vision system based on the four types of photoreceptor proteins for color: LWS, RH2, SWS2, and SWS1 (color opsins) along with RH1 (rhodopsin) for scotopic vision [2]. The opsins are photosensitive G-protein coupled receptors and are localized to outer segments of cone cells (color opsins) and rod cells (rhodopsin) in the retina, which construct pigments in the retina on binding with a polyene chromophore, retinal.

The wavelength spectrum of excitation light absorption is specific to each visual pigment. Generally, the wavelength of maximum absorbance (*λ*_max_) is ~ 500 nm in the rhodopsin RH1, while in the color opsins LWS, RH2, SWS2, and SWS1 the respective *λ*_max_ values are 510–570 nm, 470–510 nm, 440–460 nm, and 360–450 nm [2]. This mechanistic diversity enables vertebrates with all four pigment types to perceive a broad range of light stimuli, from red (longer wavelength) to near ultraviolet (shorter wavelength) radiation.

Amino acid substitutions of the photoreceptor protein responsible for *λ*_max_ are called tuning sites, which produce shifts of ~ 5 nm or more. Tuning sites have been reported in all types of visual pigments to date (e.g., [2–6]). The number of opsin genes and *λ*_max_ of visual pigments are tuned to the dominant light wavelength spectra in their habitat as the result of adaptive evolution [1]. For example, in the most diverse vertebrates, fish, opsin genes have undergone repeated duplication events and seen dramatic shifts in the *λ*_max_ of pigments, as adaptations to diverse habitats, such as water depth [7–9].

Research has established that many mammals are dichromatic [10, 11]. This is because viviparous mammals (therians: eutherians/placentals and marsupials) have only LWS and SWS1, while egg-laying mammals (monotremes) have only LWS and SWS2, i.e., therian and monotreme ancestors independently decreased the number of opsin genes to two. Early mammals ~ 200 million years ago (Mya) competed with large diurnal reptiles, such as dinosaurs, during the Jurassic period, adopting a nocturnal lifestyle while sacrificing the effectiveness of color vision in low-light hours [12].

### Sensory system in monotremes

Extant monotremes consist of only a single platypus species (*Ornithorhynchus anatinus*, the family Ornithorhynchidae) and four species of echidnas (short-beaked echidna, *Tachyglossus aculeatus,* and long-beaked echidna, *Zaglossus* spp., the family Tachyglossidae) in two genera. These species numbers are significantly lower than the total number of extant therian mammals, which number approximately 6,000 species [13]. Mammals and sauropsids (birds and reptiles) constitute a monophyletic lineage, amniotes. According to recent whole genome-based estimations, sauropsids and mammals diverged from the common amniote ancestor 300.5 Mya during the Paleozoic era, whereas monotremes and therians diverged around 180 Mya, and the platypus and echidna lineages diverged around 54.6 Mya in the early Cenozoic era [14].

Monotremes are considered “primitive” mammals due to traits shared with reptiles, such as oviparity, cloaca, and incomplete homeothermy, but echidna and platypus are quite distinct taxa; e.g., echidna is a terrestrial animal on an invertebrate diet (predominately social invertebrates) and platypus is a semi-aquatic animal that feeds on soft-shelled invertebrates [15]. Given their phylogenetic isolation, species-specific sensory adaptations in monotremes are unique among mammals. Both echidna and platypus possess bird-like beaks or bills, which have developed mechanosensory and electrosensory systems to monitor their specific environments and find prey [15]. Monotremes lead solitary lives but are seasonal breeders; therefore, olfactory and pheromonal sensory systems may have also developed functions to aid communication during breeding [14, 15]. Interestingly, the semiaquatic platypus and the terrestrial echidna rely on distinct olfactory sensory modalities. Neuroanatomical evidence indicates that platypus has the larger accessory olfactory bulb (AOB), which may be related to pheromone sensing, while echidna has the larger main olfactory bulb (MOB) [15].

Our previous analysis in monotreme genomes [14] found that this olfactory bulb difference is consistent with repertoires of chemosensory receptor genes. The number of the AOB-linked vomeronasal receptors (V1Rs) is extraordinarily higher in platypus (262) than in short-beaked echidna (28); V1R diversity may help in finding pheromones from reproductive partners, which cannot be detected by mechano- and electrosensory cues in semi-aquatic environment of platypus. In contrast, the number of MOB-linked olfactory receptors (ORs) is extraordinarily larger in echidna (693) than in platypus (299). In echidna, the electrosensory system of the beak is not as well-developed as that in platypus [15], however the larger repertoire of ORs in the echidna may help in the identification of odors from ant and termite prey underground when foraging.

The repertoire and functional properties of bitter taste receptors (TAS2Rs) also differ in platypus and echidna [16]. Platypus has seven TAS2Rs, including broad-tuned receptors that recognize a wide range of bitter compounds, whereas echidna has only three TAS2Rs, and the receptive range is narrower. These reflect the specific foraging ecologies in which platypus ingests the various repertoire of aquatic invertebrates, whereas echidna preys on colonial social ants and termitesthus necessitating less gustatory discrimination.

In the present study, we sought to characterize the visual sensory system in monotremes. To date, there have been no studies that focus on the comparison of color senses between platypus and echidna at the molecular level. As noted above, monotremes are dichromatic, with only SWS2 and LWS in their color opsin repertoires, unlike therians, which possess LWS and SWS1. Among the “tetrachromatic” ancestral repertoire (LWS, RH2, SWS2, and SWS1), RH2 was first lost as the “trichromatic” common ancestor of extant mammals, presumably due to the overlap with the receptive wavelength range of the rod pigment RH1, which was less necessary in their nocturnal environments [17]. The retention of long-wavelength reception that occurred in the LWS of all monotremes, marsupials, and eutherians is also thought to be attributable to the fact that moonlight contains a large amount of light around 560 nm, making LWS better suited to light reception at night [18]. Both SWS1 and SWS2 are responsible for short-wavelength light. In the common ancestor of marsupials and eutherians, SWS2 was lost, resulting in their repertoire of two pigments, SWS1 and LWS. In contrast, the common ancestor of platypus and echidnas lost SWS1, leaving SWS2 and LWS [11, 17].

Generally, the *λ*_max_ of SWS2 (440–460 nm) is longer than that of SWS1 (360–450 nm) [2]. We propose that SWS1 was lost in in the common monotreme ancestor as an adaptation to to its semi-aquatic habitats in rivers and lakes, as some fossil records and morphological evidence suggest that the common ancestor of platypus and echidna had a (semi)aquatic lifestyle similar to that of the extant platypus [19, 20]. Previous fish studies showed that the opsin repertoire and *λ*_max_ were changed toward longer wavelengths in fish species that inhabit cloudy rivers compared to those that inhabit the ocean or clear freshwater [21–23], possibly because long-wavelength light is abundant in highly turbid water [22]. Furthermore, semiaquatic crocodilians are thought to have had nocturnal Mesozoic ancestors, as they have lost several opsin genes and, similarly to monotremes, only have SWS2 and LWS receptors [24]. Thus, a shorter, but not the shortest, wavelength-sensitive pigment, SWS2, may have been selected in the common ancestor of extant monotremes due to visual adaptation to the light environment of rivers and lakes. Although echidnas have also been observed to swim in rivers during migration, they are fundamentally terrestrial. It is possible, but not has not been verified, that the color opsin function may have evolved differently between platypus and echidnas.

To date, only the *λ*_max_ of RH1, SWS2, and LWS of the platypus and RH1 of short-beaked echidna have been reported [17, 25]. Visual adaptations in platypus and echidnas after their divergence has been little studied. In the present study, we investigated evolutionary relationships and evaluated the *λ*_max_ difference in the color opsins of platypus and short-beaked echidna. We further analyzed 24-h behavioral data from captive short-beaked echidnas recorded at a zoo to gain a better understanding of their their visual adaptation strategy.

## Methods

### Genetic samples and sequencing

The chromosomal locations, untranslated region (UTR), and intron sequences of monotremes visual photoreceptor genes were identified in whole-genome assembly sequencing [14] using similarity search methods by the software BLAST, Splign, and MAFFT [26–28]. Monotreme photoreceptor sequences previously reported were used as search queries (Table S1) [11, 17, 25].

The genomic DNA of a platypus and a short-beaked echidna were extracted and purified from wild animals in New South Wales (NSW) and South Australia (SA), respectively. PCR amplification for all exons of LWS genes (six exons) and SWS2 (5 exons) was performed by TaKaRa Ex Taq Hot Start Version (Takara Bio) using primers designed in their UTR and intron regions (Table S2). After confirmation of PCR products by visualization of agarose gel electrophoresis, direct Sanger sequencing was performed using Big Dye Terminator v3.1 Cycle Sequencing Kit and 3130xl Genetic Analyzer (Applied Biosystems), using PCR and inner primers as sequencing primers. If there were two or more heterozygotic sites of single nucleotide variants (SNVs) within an exon, exon haplotypes were visualized and determined by additional PCR experiments using SNV-sensitive HiDi DNA polymerase (myPOLS Biotec) and primer pairs that include SNV at the last 3′-end.

### Molecular evolution

Next, using annotated sequences from whole genome sequences, sequences reported in previous studies, and newly determined in this study, phylogenetic tree construction and ancestral sequence inference were performed using the Maximum-Likelihood (ML) method using MEGA X [29]. The number of non-synonymous and synonymous sites in each sequence was calculated by PAL2NAL [30]. The *d*N/*d*S (*ω* ratio) test of each branch was performed using Fisher’s exact test [31].

### Reconstruction of monotreme visual pigments

Visual pigments were reconstructed according to the method of Kawamura and Yokoyama [32]. Based on the determined nucleotide sequences of opsin haplotypes, expression vectors were artificially synthesized (FASMAC). The insert nucleotide sequences were confirmed by Sanger sequencing after miniprep purification. The purified plasmid vector was transfected into COS-1 (transformed green monkey kidney fibroblast cell). 11-*cis* retinal was mixed with the suspension. Reconstructed visual pigments were purified using Sepharose beads with Rho1D4 antibody.

### Absorbance spectral analysis

Absorbance spectra of reconstructed visual pigments were measured using a U-3010 spectrophotometer (HITACHI) at 20 °C under dimly lit, red-light conditions. Scan ranges were 250–700 nm in SWS2 and 250–750 nm in LWS. Scan speed was set to 300 nm/min, slit width to 0.5 nm, and cell length to 10 nm. After the dark spectrum was measured eight times, visual pigments were bleached by irradiating a 100W filament lamp filtered 440 nm and less for 3 min. Thus, the light spectrum was measured eight times using bleached pigments.

In order to visualize the measured spectra, cubic smoothing spline curves were fitted to the mean of dark, light, and difference (dark minus light) spectra of each pigment, using “smooth spline” in the statistics of the package R (spar = 1; all.knots = TRUE). *λ*_max_ was estimated from the mean of the peak of each spectrum. The peak of the dark spectrum of the analyzed LWS could not be identified. Therefore, *λ*_max_ was calculated from the difference spectrum (dark spectrum minus light spectrum) according to Kawamura and Yokoyama [32]. The represented value of *λ*_max_ and its standard deviation were calculated based on the eight-time measurement.

### Captive echidnas

The behavior was recorded from two captive short-beaked echidnas at the Nagoya Higashiyama Zoo (Nagoya, Japan), named “Shou” and “Dai.” They were introduced from Adelaide Zoo (South Australia) with one additional individual (now deceased) in 1977. Their precise ages and sexes are unknown, but they were over 45 years old at the time of the study, while their geographical origin was estimated using mitochondrial DNA phylogeny. For genetic analysis, spines that were naturally shed from each echidna and collected non-invasively by caretakers were used. After an ethanol rinse, genomic DNA was extracted and purified from ~ 1 cm of the root end of a spine using the QIAamp DNA Investigator Kit (QIAGEN). PCR amplification and Sanger sequencing of the partial D-loop region of the mitochondrial genome were performed using a pair of TacCRF and TacCRR [33]. The D-loop sequences of “Shou” and “Dai” are identical both to each other and to a haplotype discovered in NSW and Queensland (QLD), suggesting that their maternal origin is eastern Australia.

Behavioral data collection was performed from 28 November to 27 December, 2021. These two echidnas had only been cared for in an in-house enclosure (300 cm wide, 200 cm deep, and ~ 200 cm high) together with each other. The ambient temperature of the enclosure was programmed at approximately 25–30 °C by automatic air conditioning equipment, which is a comparatively warm environment for echidna [34]. The ground was covered with fine sand with rock-like objects made of mortar. An infrared lamp was placed in the corner of the enclosure and lit all the time, providing an uninterrupted warm area for the echidnas. From 10:47 pm to 9:47 am (11 h), the white fluorescent lamp was lit in all the enclosure areas during the light condition (light hours). Then, from 9:47 am to 10:47 pm (13 h), the white, fluorescent lamp was extinguished at dark condition (dark hours).

In most cases, caretakers clean the enclosure for ~ 5 min between 8:30 am and 9:30 am and leave a new feed tray in a moment from 11:30 am to 12:30 pm. Caretakers kept entry to the enclosure and interference with echidnas to a minimum in terms of animal welfare. The enclosure and the viewing area were separated by transparent glass. Both echidnas and visitors are visible to each other via the glass and an share a more or less identical audial environment. Visitors can enter the viewing area from 9:00 am to 4:45 pm, mostly accounted for by night. The zoo was closed on Monday.

### 24-h behavior recording and ethogram

A wide-angle camera (GoPro HERO7 Black; GoPro, Inc.) was equipped on the ceiling of the enclosure so that the entire enclosure could be filmed. Timelapse photography (0.5-s interval) was continuously recorded for 609 h on 27 days (28 November to 18 December and 22–27 December, 2021).

First, echidna behavior was classified as “active” and “nonactive” each 1 min. “Active” indicates echidnas showed any behavioral change in a given 1 mi, while “nonactive” indicates echidnas showed no reactive response, i.e., only resting or sleeping. Observations of the time-lapse series of “active” minutes at a glance were made to assemble an ethogram (data collection of quantifiable behaviors). It was presumed that there were external factors that potentially affect echidna behaviors. For example, light–dark changes may affect visual-oriented behaviors. Human interference, such as feeding and enclosure cleaning by caretakers and visitors’ gaze and sounds, may also affect echidna behaviors. Statistical analysis was performed using R software (https://www.r-project.org/).

## Results

### Chromosomal location of visual photoreceptors

The short-beaked echidna genome has 27 autosomes, five X, and four Y chromosomes, whereas the platypus genome has 21 autosomes, five X, and five Y chromosomes [35]. The monotreme genome project recently released a whole-genome assembly at the chromosome level of the echidna and platypus [14]. Our BLAST-based similarity searches found that in both echidna and platypus, the LWS, SWS2, and RH1 genes are located on chromosomes 6, 6, and X1 with six, five, and five exons, respectively (Table S1). The tandem arrangement of LWS and SWS2 in the same autosome is consistent with previous studies; this synteny is shared among many vertebrates [11, 36].

Davies et al. (2007) [17] predicted that monotreme LWS might be X-linked, as in therians, due to their evolutionary relationships, but this possibility was rejected in the present study. Monotreme LWS is located on chromosome 6. In contrast, as a new finding, the monotreme RH1 (rhodopsin) is located on the sex chromosomes X1. X-linked visual photoreceptors have not been known except for therian LWS [37]. Uniquely in monotremes, the five pairs of sex chromosomes are thought to be constructed by incorporating ancestral autosomes into the sex chromosome chain. Chromosome-scale synteny analysis by Zhou et al. [14] showed that monotreme chromosome X1 is homologous to parts of human chromosome 3 and chicken chromosome 12, where human and chicken RH1 are located there, respectively. Therefore, monotreme RH1 appeared to be incorporated into chromosome X1 at the chromosomal scale, not as a small-scale translocation.

### SNVs, haplotypes, phylogeny, and molecular evolution

Coding sequences of all opsins for color vision (LWS and SWS2) were determined in wild monotremes, a platypus from NSW, and a short-beaked echidna from SA. All gene sequences were intact. The platypus did not have a heterozygotic single-nucleotide variant, whereas the echidna had heterozygotic SNVs in both LWS and SWS2. The SNV sites of the LWS of the echidna were in the second and sixth exons. Interexon linkages are unknown, but a haplotype of the first to sixth exon each was identical to that of a NSW echidna (CM027604.1). Therefore, one of the LWS haplotypes of the SA echidna was regarded as identical to the NSW echidna (Table S3).

**Fig. 1:**
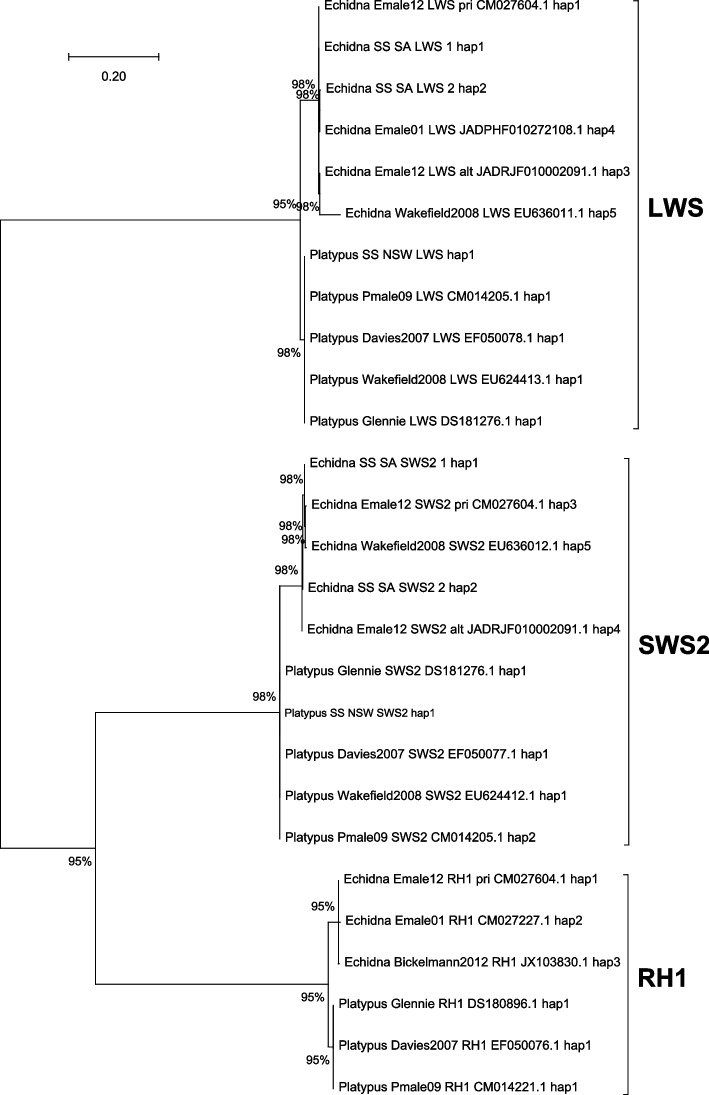
A gene tree of 27 of the 28 identified opsin sequences is shown. One sequence (Emale01 SWS2) is truncated and is not included. This tree is constructed with the highest log likelihood inferred by using the Maximum Likelihood (ML) method and Hasegawa-Kishino-Yano (HKY) model. The best model (HKY + G + I) with the lowest Bayesian Information Criterion (BIC) score was considered to describe the substitution pattern the best. The percentage of trees (1000 bootstrap replications) is shown next to the branches. The initial tree(s) for the heuristic search were automatically obtained by Neighbor-Join and BioNJ algorithms (five Gamma categories)

Combined new findings with those from with previous studies and whole genome sequences, we identified a total of 28 phased available visual photoreceptor nucleotide sequences. The number of nucleotide haplotypes in visual opsins seemed to be higher in echidna (3–5 haplotypes in each gene) than that of platypus (one or two haplotypes) (Fig. 1). The haplotype diversity of the visual photoreceptors seemed to be higher than that in platypus.

**Fig. 2:**
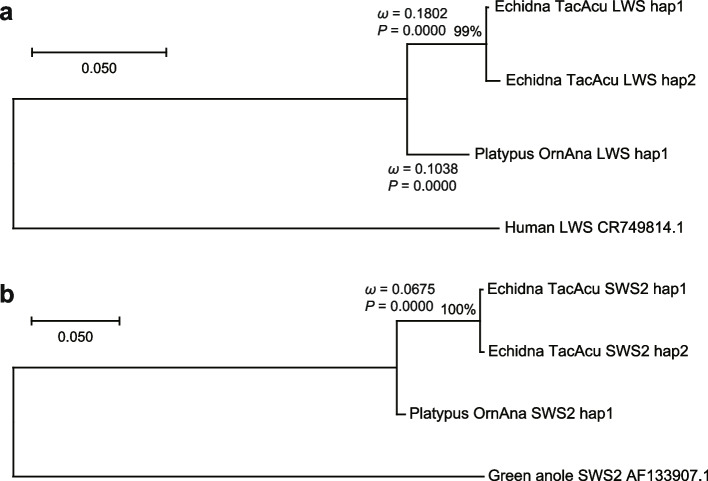
Results of the *d*N/*d*S (*ω*) test of each branch (between the common ancestor of echidna and platypus and each species) in the monotreme color opsin genes. The best tree topology and ancestral sequences were estimated by the ML method. The trees were routed by ortholog genes of non-monotreme amniotes. Statistical tests were performed using Fisher’s exact test. **a** LWS gene. **b** SWS2 gene. OrnAna SWS2 hap1 corresponded to the ancestral sequence; *ω* of the branch could not be calculated

Based on identified haplotypes, ML tree construction, ancestral sequence estimation, and synonymous/non-synonymous site calculation were performed (Fig. 2a: LWS; Fig. 2b: SWS2). Both branches of the platypus and echidna lineages showed that *ω* < 1 (*P* < 0.0001), indicating that the color opsin genes are subject to purification in both monotreme species. The platypus and the last common ancestor of platypus and echidna had identical SWS2 at the amino acid level, indicating that the molecular evolution of the SWS2 gene in platypus was especially conservative.

Yokoyama et al. [3] proposed the five-site rule in LWS, where the five amino acid residues are responsible for *λ*_max_ as tuning sites (Table S4). The corresponding five sites are S180, H197, Y277, T285, and A308 in echidna LWS, while A180, H197, Y277, T285, and A308 in platypus, i.e., only site 180 differed between echidna and platypus. The echidna S180 was the ancestral amino acid state, and there were no intraspecific differences at the five sites. The estimated *λ*_max_ based on the five-site rule, platypus LWS *λ*_max_ is 555 nm, while echidna LWS *λ*_max_ is 560 nm. A previous study of fish in SWS2 revealed that amino acid residues 91, 94, 116, 122, 261, 292, and 295 (in the numbers of fish in SWS2) were responsible for ~ 5 nm or more shift of *λ*_max_ [4, 5]. However, there were no differences between platypus and echidna SWS2 in these residues (Table S5).

### Absorption spectrum and *λ*_max_

**Fig. 3:**
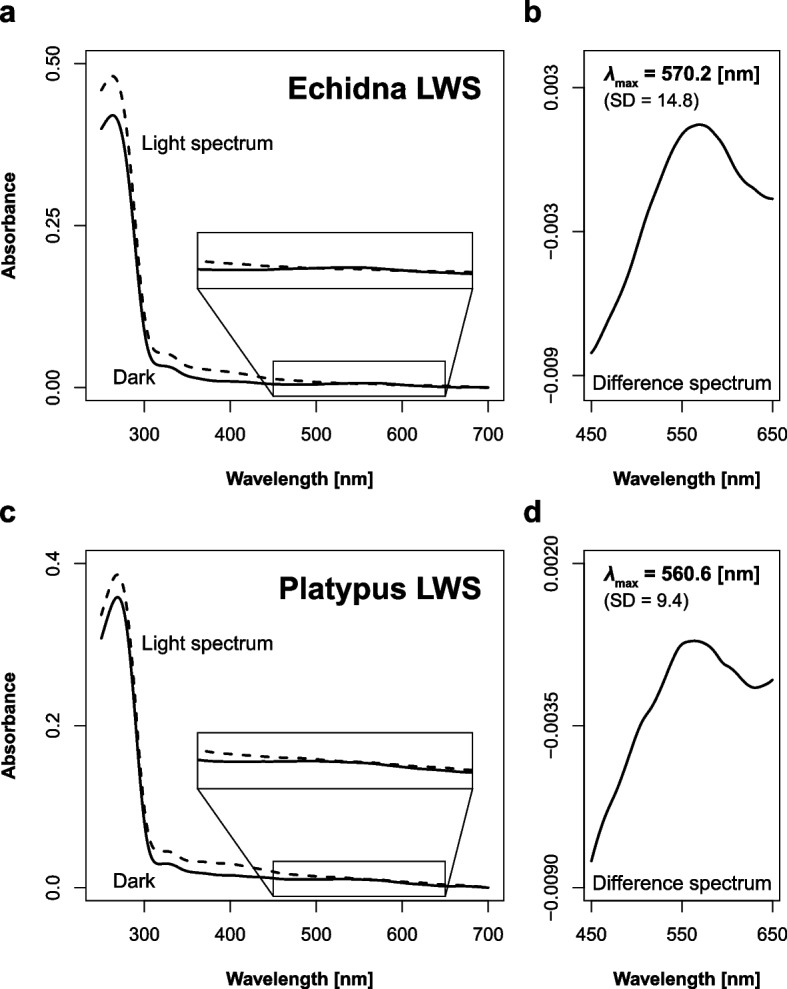
Absorption spectra of monotreme LWS opsins. Dark spectra and difference spectra of echidna **a**,** b** and platypus **c**,** d** are shown, respectively. *λ*_max_ could not be calculated in dark spectra of monotreme LWS due to low absorbance. Light and dark spectra of 450–650 nm are enlarged to highlight the absolute spectra around the *λ*_max_

**Fig. 4:**
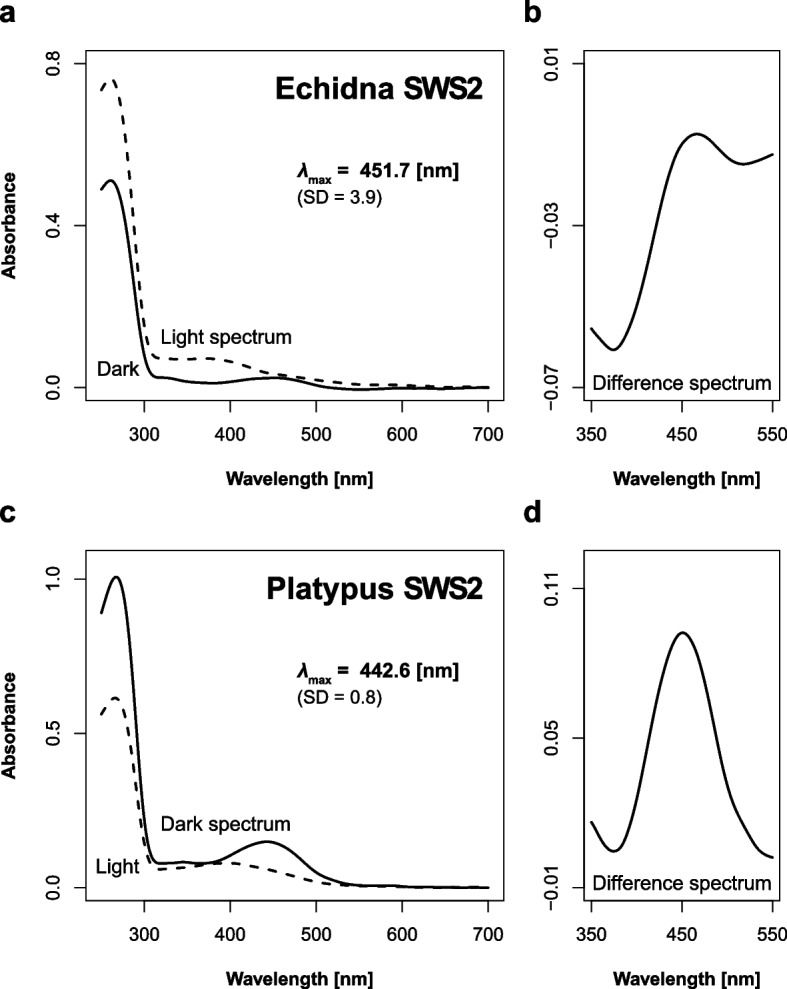
Absorption spectra of monotreme SWS2 opsins. Dark spectra and difference spectra of echidna **a**,** b** and platypus **c**,** d** are shown, respectively

We were able to obtain absorption spectra from all the cone opsins analyzed in this study. Basically, *λ*_max_ is measured directly from the dark spectrum, but peaks were not detected from the LWS dark spectrum in either echidna or platypus, due to low absorbance (Fig. 3). As an alternative, *λ*_max_ of the difference spectrum was measured, yielding 570.2 ± 14.8 nm and 560.6 ± 9.4 nm (mean ± standard deviation) in the LWS of echidna and platypus, respectively (Fig. 3). *λ*_max_ of the dark spectra in SWS2 were measured, 451.7 ± 3.9 nm in the echidna and 442.6 ± 0.8 nm in the platypus (Fig. 4). In both LWS and SWS2, the echidna has ~ 10 nm longer *λ*_max_ than the platypus, i.e., the spectral range in the echidna color vision is relatively shifted overall compared to platypus (*P* = 0.15 for LWS and *P* = 0.00026 for SWS2, Welch’s *t*-test).

A previous study [17] reported that platypus *λ*_max_ in difference spectra were 550 nm in LWS and 451 nm in SWS2, which our results indicate exhibits a ~ 10 nm difference. Although the previous study did not report standard deviation, our platypus LWS *λ*_max_ in the difference spectrum shows a large standard deviation: 560.6 ± 9.4 nm. The inferior end was 551.2 and this is very close to that reported in the previous study (550 nm). Therefore, this inconsistency is thought to be due to an experimental error. For SWS2, we directly observed the dark spectrum but the previous study used the difference spectrum as an indirect observation. In short-wavelength sensitive opsins (i.e., SWS1 and SWS2), distortion between light and dark spectra is not ignorable. For example, estimated *λ*_max_ in SWS2 of American chameleon (*Anolis carolinensis*) was 437.0 ± 1.6 in the dark spectrum and 452.3 ± 0.5 in the difference spectrum [32]. Therefore, the ~ 10 nm difference in platypus SWS2 between our study (442.6 ± 0.8, dark spectrum) and the previous study (451, difference spectrum) may be attributable to a difference in calculation methods.

Bovine RH1 (rhodopsin) was used as a positive control. In order to culture the COS-1 cells integrated with bovine RH1, five Petri dishes (15 cm in diameter) were used, leading to a clear dark spectrum and prominent peak (*λ*_max_ = 498.8 nm) (Fig. S1). The ratio of maximum absorbance at around 280 nm (protein peak) to absorbance at *λ*_max_ was 3:1. In contrast, 20 Petri dishes were required to culture monotreme SWS2. Nevertheless, the maximum absorbance at the protein peak was about half as low as bovine RH1, and the ratio is 10:1 (Fig. 4). Furthermore, monotreme LWS requires more than 30 Petri dishes, but the maximum absorbance at the protein peak was about half that of low bovine RH1, and the *λ*_max_ peak was quite low (Fig. 3). Although the reason cause of the poor expression efficiency and absorbance level is unclear, similar findings have been reported in many other vertebrates [32, 39], and the results were found to be reliable.

### Daily activity of echidnas

**Fig. 5:**
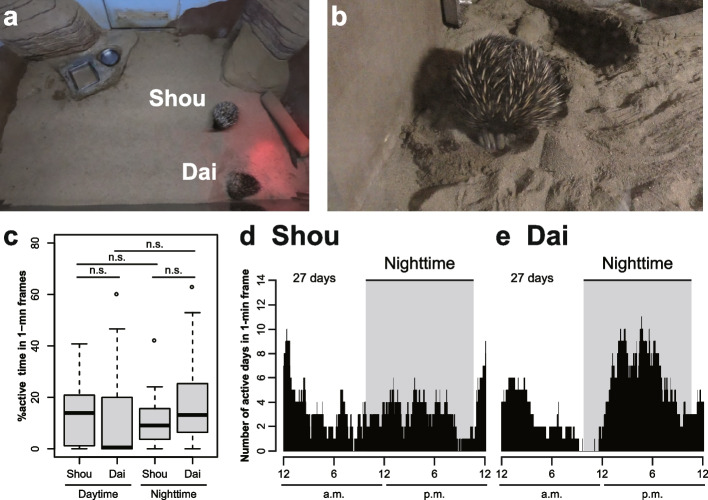
Twenty-four-hour activity patterns of two captive echidnas (named “Shou” and “Dai”) in the same enclosure (609 h of 27 days) at a stable temperature (25 °C). **a** A snapshot of time-lapse photography from a wide-angle camera, positioned to enable filming of the entire enclosure of the two echidnas (Shou and Dai). **b** A behavioral example. Dai digs in the sand. **c** Box plots of the frequency of active time in comparison between the light and dark conditions and among individuals. There were no significant differences (Wilcoxon signed rank tests with Bonferroni correction, *P* > 0.05). Histograms of the 24-h activity pattern in (**d**) Shou and (**e**) Dai

The activity (active or nonactive) times of two captive echidnas living together (Shou and Dai) were analyzed using time-lapse for a total of 609 h over 27 days (Fig. 5a, b). In general, they were active during 24-h of each day (Fig. 5c). Shou showed the highest peak of activity at around 12:00 am, around 1 h after the day started (10:47 pm) (Fig. 5d). However, Dai showed the highest peak of activity at around 3:00 pm, the middle of the night (Fig. 5e). Thus, activity time has individual-specific patterns.

**Fig. 6:**
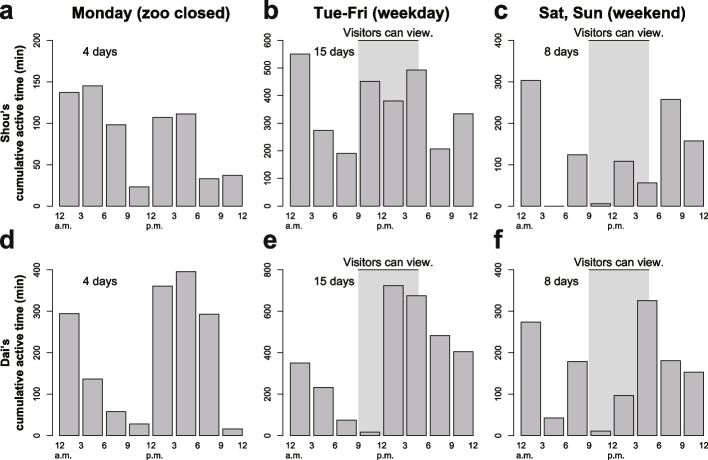
Total activity time in terms of visitors’ effect (**a–c**: individual “Shou”; **d–f**: individual “Dai”). The studied Japanese zoo was closed on Mondays (**a**,** d**). In Japan, it was a weekday from Tuesday to Friday (low visitor numbers) (**b**,** e**), and weekends (Saturday and Sunday, high visitor numbers) (**c**,** f**). The activity times were statistically different among these three groups according to chi-squared tests (Shou: *N* = 4581, *χ*^2^ = 866.72, df = 14, *P* < 2.2E^−16^; Dai: *N* = 5810, *χ*^2^ = 663.23, df = 14, *P* < 2.2E.^−16^)

Whether characteristic behavioral sequences occurred or not after caretakers cleaned and fed was surveyed, and such sequences were not observed. The activity was compared from Monday (zoo closed), Tuesday to Friday (weekday), and Saturday and Sunday (weekend) (Fig. 6). As a result, the activity time was statistically different among these three groups, as shown by chi-squared tests (Shou: *N* = 4581, *χ*^2^ = 866.72, df = 14, *P* < 2.2E^−16^; Dai: *N* = 5810, *χ*^2^ = 663.23, df = 14, *P* < 2.2E^−16^), suggesting that visitors’ viewing behaviors and noise affected echidna activity. Notably, the two echidnas did not show evident interactions at the behavioral level.

### Ethogram

**Fig. 7:**
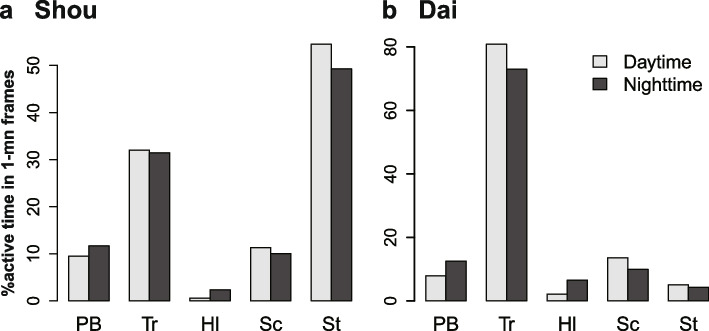
Ethogram-based frequency of each behavioral category in comparison between the light and dark conditions. **a** individual Shou. **b** individual Dai. Abbreviations: PB, Primary behavior; Tr, Traveling; Hl, Halting; Sc, Searching; St, Self-treatment

The behaviors that were frequently observed were classified as “primary behavior,” “traveling,” “halting (terminating other active behavior),” “searching,” and “self-treatment” (Fig. 5b; Table 1). Based on the ethogram, the number of 1-min active frames of each behavioral category was counted (Fig. 7). Since preliminary analysis indicated that frequent behavioral changes occurred within a frame, counting multiple behavioral categories was allowed, which provided near continuous activity budget data. Among classifiable behaviors, the most frequent behaviors were “self-treatment” by Shou (Fig. 7a) and “traveling” by Dai (Fig. 7b). Generally, the frequency of each behavior did not have a noticeable difference between the light and dark conditions, except “halting.” “Halting” behaviors were more frequent at night in both individuals.

**Table 1:** List of ethogram in short-beaked echidna

	**Definition and example**
Primary behavior	Actions including feeding, drinking, and defecation
Traveling	Only traveling
Halting	Actions of stopping oneself from active states that continue for 10 min or less
Searching	Actions including looking around, sniffing, licking, digging, and moving objects using the beak
Self-treatment	Actions including self-grooming using limbs, stretching, inspecting abdomen, and self-immersion in water

## Discussion

### Cooperative spectrum shift in LWS and SWS2 in echidna and platypus

Molecular evolution analysis showed that LWS and SWS2 in echidna and platypus were subject to strong functional constraints. *d*N/*d*S (*ω*) of LWS and SWS2 were significantly less than 1, indicating purifying selection. Furthermore, the platypus and the echidna common ancestor presumably had identical SWS2 at the amino acid level, indicating that the evolution of the platypus SWS2 gene was especially conservative. *λ*_max_ has ~ 10-nm differences between echidna and platypus in both LWS and SWS2, indicating that echidna and platypus ancestors obtained a few functional amino acid changes and that strong purifying selection occurred and continued.

The five-site rule of LWS adjusts its *λ*_max_ to the range of 510–560 nm [3]. The estimated *λ*_max_ by the five-site rule was 560 nm in the common ancestor and echidna, and 555 nm in platypuses. This 5-nm interspecific difference is due to the S180A amino acid substitution. Echidna and the last common ancestor of echidna and platypus has S180 (presumably ancestral state) and platypus has 180A (presumably derived state) (Table S4). However, spectroscopically measured *λ*_max_ from difference spectra of reconstructed pigments were ~ 10 nm longer than the five-site rule estimation and had ~ 10-nm difference between echidna and platypus, i.e., 570.2 nm in echidna and 560.6 nm in platypus. Consistently with the five-site rule, the LWS *λ*_max_ is longer than the platypus. More unknown residue(s) as well as S180A substitution must affect the species difference.

In other vertebrates, the human paralogs, MWS (*λ*_max_ = 530 nm) and LWS (*λ*_max_ = 560 nm) have 30-nm differences. Zebrafish also have paralogs, LWS-1 (*λ*_max_ = 558 nm) and LWS-2 (*λ*_max_ = 548 nm), which have a 10-nm difference [38, 40]. The *λ*_max_ of SWS2 also showed the ~ 10-nm difference spectroscopically between echidna and platypus, i.e., 451.7 nm in echidna and 442.6 nm in platypus.

Interestingly, LWS and SWS2 of echidna *λ*_max_ were longer than those of platypus. We hypothesize that cooperative spectrum shifts in LWS and SWS2 by the same amount of ~ 10 nm and in the same direction might occur in echidna and platypus. In many aquatic mammals, freshwater habitats require short-wavelength sensitive vision. Extinct toothed platypuses, such as *Obdurodon*, are hypothesized to have been pelagic foragers in non-cloudy waters [41], and thus may have benefited from comparatively shorter wavelength color vision. Since the ancestral platypus obtained the substitution S180A, which makes *λ*_max_ shorter, this substitution would contribute to its life in non-cloudy areas. Extant platypuses do not use vision in their aquatic behaviors as they close their eyes and use their electrosensory system in cloudy water [41]. Extant platypuses have continued their terrestrial activity such as breeding. S180A in extant platypuses as well as ancestral toothed platypuses may also help their limited terrestrial behavior.

The terrestrial adaptation in echidna likely requires long-wavelength color vision to identify various environmental light conditions throughout Australia and Papua, but further analyses are required, such as population-level polymorphic analysis. Our wild sample of South Australian echidna was heterozygotic with SNVs. We believe that a more extensive sampling of short-beaked echidnas will help determine visual adaptations to the diverse habitat.

This study is not focusing on functional variations within species, but intraspecific visual sense variations often occur under the balancing selection. Functional variations of LWS and its duplication within species in primates, including humans, lead to sex-biased red-green color vision based on sex [42]. However, we found that monotreme LWS is not located on chromosome X, but rather on chromosome 6, contrary to the previous expectation [17]. Sex-biased color vision cannot occur in monotremes. Interestingly, our findings revealed that monotreme RH1 (rhodopsin) was X-linked. There were multiple haplotypes with non-synonymous substitution in echidna RH1 samples in this study. Although it is unknown whether there are intraspecific functional variations of RH1 in monotremes, as has been reported in primate LWS. Thus, further analysis of the functional mechanism of X-linked rhodopsin visual sensory systemin monotremes would also be of interest.

### Light use in short-beaked echidnas

Light environments and availability have a strong influence on animal activity. The spectra of daylight in the daytime, twilight in the evening, and moonlight at night are quite different from each other [18, 43]. The light spectra of freshwater and cloudy water differ, and water depth also affects spectra and intensity [21–23]. Color opsins are functionally constrained or relaxed by these light environments of habitat. For example, the nocturnal aye-aye, which uses short wavelengths in the evening, shifted their SWS1 *λ*_max_ to ~ 400 nm, although *λ*_max_ of SWS1 in many primates is ~ 430 nm [44]. Moonlight also includes short-wavelength light. In nocturnal, arboreal lemurs (primates of Madagascar), species from the open canopy forest experienced strong purifying selection in maintaining SWS1, whereas species from open canopy forest, which are impoverished in short wavelength light, experienced relaxed selection [45]. Population structure and fluctuation also influence the opsin genetics. The decrease of intraspecific nucleotide diversity in X-linked LWS of a wild population of red-bellied lemurs (*Eulemur rubriventer*) resulted from a possible specific situation of the recent genetic bottleneck [46].

We sought to determine whether the platypus and echidna are diurnal, nocturnal, crepuscular, cathemeral, or otherwise. In the literature, they are sometimes described as diurnal and sometimes as nocturnal [15, 34]. In particular, captive echidnas in Japanese zoos, including our studied individuals, were traditionally displayed alongside nocturnal animals. The step-by-step loss of color opsins, from four to three in early mammals and from three to two in early monotremes may be related to nocturnal activity. Twenty-four-hour activity studies in monotremes have been limited. Several field studies reported that wild echidna and platypus are active both during the day and at night and that, moreover, their activity time was dependent on ambient temperature [34, 47, 48]. For example, wild echidnas in a semi-arid area of western Australia showed a marked seasonal change in foraging activity from mostly nocturnal during the hot months to afternoon and night in winter [34].

As a characteristic physiology of monotremes, their body temperatures fluctuate daily due to incomplete homeothermy [15, 34]. Routine occurrences of daily torpor and long-term hibernation were observed in short-beaked echidna, not platypus and long-beaked echidna [49]. Therefore, in free-ranging conditions, their activity patterns strongly depend on fluctuations in ambient temperature. Moreover, these activity studies were mainly based on radio-tracking and data logging. Quantitative behavioral analysis based on direct behavior recoding and ethogram like our current study was performed yet. Our studied captive echidnas in Higashiyama Zoo, Japan, were under stable temperature (25 °C). Nevertheless, these echidnas were active for 24 h. The behavioral repertoires were not different between the day and night hours. These suggest that daily fluctuation of ambient temperature does not necessarily affect its strict cathemerality (active in both light and dark conditions).

Ethogram-based analysis showed that “halting” behavior was more frequent in the dark condition than the light condition (Table 1; Fig. 7). This may be because echidnas are visually more careful and use other sensory information to guide behaviors in the dark condition. Since echidnas also use this highly developed olfactory sensory system [14], they can use olfaction during dark hours, an alternative to the color vision used in light hours. The cathemeral echidnas may be more dependent on vision during the day and olfaction at night.

### Usage of color vision in echidna and platypus ancestors

Our molecular evolution and spectral analysis indicated that the selective pressure of LWS and SWS2 was very strong, and the spectral range cooperatively shifted, ranging ~ 10 nm in the evolution from the last common ancestor of echidna and platypus to the extant echidna and platypus. However, the biology between echidna and platypus is clearly quite different, and their ~ 60-million-year evolutionary trajectory is very dynamic. Thus, we sought to determine how the LWS and SWS2 features have worked in the monotreme evolution.

Chromosomal-scale genome analysis of short-beaked echidna and platypus showed that the divergence time of echidna and platypus is 54.6 Mya [14]. The oldest platypus fossil (*Monotrematum*) is dated to 63–61 Mya [20]. Therefore, the last common ancestor of echidna and platypus likely occurred in ~ 60 Mya. The following platypus fossil (*Obdurodon*) is 26–13 Mya [20]. *Obdurodon* and extant platypus are believed to use different sensory systems and employ different foraging strategies. Fossil evidence suggests that *Obdurodon* was aquatic or semi-aquatic, had less developed electroreceptive capacities, and foraged in the water column (surface of rivers and lakes) with its eyes open [41]. Color vision enabled by LWS and SWS2 genes would help them forage in the water column without relying on non-visual senses, such as an electrosensory system. Extant platypuses have maintained their semiaquatic life, but in stark contrast, they forage diet in cloudy water at the bottom of streams and lakebeds with their eyes closed, which situation does not enable them to use color vision, and alternatively use well-developed electroreceptive capacities. The existing platypuses use the land for nesting and reproduction, but their olfaction is not well-developed, in contrast to extant echidnas [14], suggesting that LWS and SWS2 from *Obdurodon*’s era aided in terrestrial activities during the day and were perhaps adapted to use in twilight and moonlight.

Additionally, we considered the origin of the echidna family Tachyglossidae. Whether early echidna ancestors ~ 60 Mya (starting the Cenozoic era) were (semi-)aquatic like platypus are still debated [19, 20, 50]. No Paleogene (early Cenozoic) echidna fossils are currently unknown. Therefore, the biology of early echidna do not have paleontological consensus, unlike the platypus family Ornithorhynchidae. *Megalibgwilia* recognized as one of the oldest echidnas, may occur in the Neogene period (middle Cenozoic), but exhibit similar traits to those of extant short-beaked and long-beaked echidnas, such as predation on terrestrial invertebrates [20]. Current knowledge cannot tell us about the spectral range of SWS2 and LWS in echidna ancestors, but SWS2 and LWS of the echidna lineage after split from the platypus lineage, which trends to longer wavelengths than the extant platypus and strong selective constraints, would contribute to their terrestrial lifestyle.

### Toward conservation and welfare

We first reported comprehensive 24-h behavioral collection and ethogram in echidna, suggesting that their cathemerality does not necessarily depend on temperature fluctuation. As neuroanatomical and genomic analysis [14] showed, echidna has developed olfactory system. Ethograms qualitatively showed that echidnas exhibit smelling behaviors (Table 1). Color vision in the day and olfaction at night are used in echidnas’ terrestrial life. Although limitations remain in terms of the captive condition and sample size, our ethogram and 24-h data collection will lead to new understanding echidna sensory behavior and ecology.

Our new findings are also relevant for the conservation of habitat for echidna and animal welfare in captivity and provide novel insight into the basic biology of short-beaked echidna. Natural populations of short-beaked echidna are stable and are less concerned about the risk of extinction but are threatened by recent catastrophic environmental changes such as urbanization and bushfires [51, 52]. Geographical variations and absorption spectra in echidna color opsins have not been reported to date. The fact that the functional constraint and abundant behavioral repertoire under daylight, as well as twilight and moonlight, indicate the reliance on color vision tells us the importance of 24-h light environment conservation of their various habitats in Australia and Papua.

Traditional monitoring of echidna behavior has been based on implant radio-tracking and data-logging. Our trial sought to construct a comprehensive ethogram and describe the 24-h activity budget in short-beaked echidnas that will be useful for behavioral enrichment and the improvement of the captive environment in captive facilities such as zoos and direct observation of wild ones. The warm temperature for the echidnas (~ 25–30 °C) led to the cathemeral activity of the captive echidnas, suggesting that to allow the captive echidnas to change to nocturnality, torpor, and perhaps reproduction success, a more artificial fluctuation of ambient temperature is very important. We also observed changes in the activity of captive echidnas in the presence of visitors (Fig. 6). We have begun environmental enrichment of the old echidnas studied here in the Higashiyama-zoo enclosure by the installation of objects, such as termite mound-like feeders to enhance their behaviors using ethogram-based observation monitoring. We believe that our study will help *ex-situ* (outside the natural habitat, especially in foreign countries) conservation and animal welfare of this mysterious egg-laying mammal.

## Conclusions

Based on our new findings, namely different *λ*_max_ in visual pigments and cathemeral daily activity of captive short-beaked echidna, we propose that (1) the common ancestor of echidna and platypus in early Cenozoic was aquatic or semiaquatic and used its dichromatic sensory system (LWS and SWS2) for foraging in the comparatively clear water column; (2) extant platypus, whose strategy was shifted from water column foraging to pelagic foraging in cloudy water using a well-developed electrosensory and mechanosensory system alternative to color vision, still uses their color vision in land life such as nesting and reproduction, and that (3) extant echidna, whose life shifting from (semi-)aquatic to terrestrial, still use their color vision with a well-developed olfactory sensory system for various 24-h terrestrial behaviors including foraging social insect and other invertebrates under daylight, twilight, and moonlight. More global population-level analyses in both wild and captive echidnas and platypuses is required, but our findings and suggestion provide new insights into the ecology and evolution and habitat conservation of egg-laying mammals’ animal welfare.

### Supplementary Information

**Additional file 1:** **Fig. S1.** Absorption spectra of bovine RH1.

**Additional file 2:** **Table S1.** Chromosomal location of visual photoreceptor genes in echidna and platypus. **Table S2.** PCR primers for LWS and SWS2 genes in echidna and platypus. **Table S3.** Nucleotide sequences and haplotypes of visual photoreceptor genes in echidna and platypus. **Table S4.** Amino acids of key site residues of the five-site rule in monotreme LWS. **Table S5.** Amino acids of key site residues in monotreme SWS2.

## Data Availability

The newly determined opsin sequences were deposited in the DDBJ database with accession numbers LC780178–LC780183.
