# Supplementary figures and images for: Color vision evolution in egg-laying mammals: insights from visual photoreceptors and daily activities of Australian echidnas
Source: Zoological Lett. 2024 Jan 2;10:2. doi: 10.1186/s40851-023-00224-7 (PMC10759620; doi:10.1186/s40851-023-00224-7)

**Fig. S1**

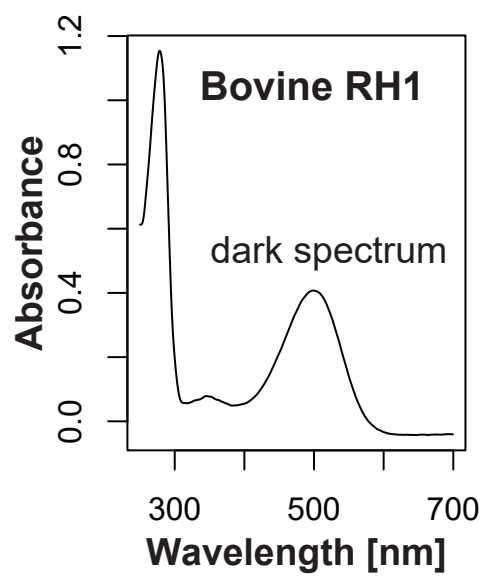

Supplement: Supplementary file 1 — Additional file 1: Fig. S1. Absorption spectra of bovine RH1. [file 40851_2023_224_MOESM1_ESM.pdf]
